# Activation of DNA Damage Response Pathways during Lytic Replication of KSHV

**DOI:** 10.3390/v7062752

**Published:** 2015-06-05

**Authors:** Robert Hollingworth, George L. Skalka, Grant S. Stewart, Andrew D. Hislop, David J. Blackbourn, Roger J. Grand

**Affiliations:** 1 School of Cancer Sciences, the College of Medicine and Dentistry, University of Birmingham, Birmingham B15 2TT, UK; E-Mails: rxh291@student.bham.ac.uk (R.H.); gls210@student.bham.ac.uk (G.L.S.); g.s.stewart@bham.ac.uk (G.S.S.); a.d.hislop@bham.ac.uk (A.D.H.); 2 School of Biosciences and Medicine, University of Surrey, Surrey GU2 7XH, UK; E-Mail: d.blackbourn@surrey.ac.uk

**Keywords:** Kaposi’s sarcoma-associated herpesvirus, KSHV, DNA damage response, DDR, lytic replication, ATM, ATR, DNA-PK

## Abstract

Kaposi’s sarcoma-associated herpesvirus (KSHV) is the causative agent of several human malignancies. Human tumour viruses such as KSHV are known to interact with the DNA damage response (DDR), the molecular pathways that recognise and repair lesions in cellular DNA. Here it is demonstrated that lytic reactivation of KSHV leads to activation of the ATM and DNA-PK DDR kinases resulting in phosphorylation of multiple downstream substrates. Inhibition of ATM results in the reduction of overall levels of viral replication while inhibition of DNA-PK increases activation of ATM and leads to earlier viral release. There is no activation of the ATR-CHK1 pathway following lytic replication and CHK1 phosphorylation is inhibited at later times during the lytic cycle. Despite evidence of double-strand breaks and phosphorylation of H2AX, 53BP1 foci are not consistently observed in cells containing lytic virus although RPA32 and MRE11 localise to sites of viral DNA synthesis. Activation of the DDR following KSHV lytic reactivation does not result in a G1 cell cycle block and cells are able to proceed to S-phase during the lytic cycle. KSHV appears then to selectively activate DDR pathways, modulate cell cycle progression and recruit DDR proteins to sites of viral replication during the lytic cycle.

## 1. Introduction

Kaposi’s sarcoma-associated herpesvirus (KSHV) is a γ-2 herpesvirus identified as the etiological agent of several human malignancies. Transformation of endothelial cells by KSHV is associated with the development of Kaposi’s sarcoma (KS) [1], while infection of B cells can lead to the lymphoproliferative diseases primary effusion lymphoma (PEL) and multicentric Castleman’s disease (MCD) [2,3].

Like all herpesviruses, KSHV has a double-stranded DNA genome and a biphasic lifecycle that consists of latent and lytic stages. During latent infection, the virus persists as an extrachromosomal episome that is replicated by host replication machinery. Latency is characterised by expression of a limited subset of viral genes required for episomal maintenance and immune evasion that include the latency-associated nuclear antigen (LANA). In order to propagate, the virus must switch to the lytic phase, during which the majority of viral genes are expressed, viral DNA is amplified and infectious virus is released following lysis of the host cell. The viral replication and transcription activator (RTA), encoded by open reading frame 50 (ORF50), is a lytic switch protein as expression is necessary and sufficient for initiation of the full lytic replication programme [4]. Lytic viral genes are expressed in an ordered cascade and the lytic cycle can be divided into several stages that include immediate-early gene expression, delayed-early gene expression, viral DNA amplification, late gene expression and finally, assembly and release of infectious virions [5]. Significantly, lytic replication has been shown to play a major role in the progression of KSHV-related malignancies [6,7].

The DNA damage response (DDR) has evolved to respond to the multitude of lesions inflicted on cellular DNA by both endogenous biological processes and exogenous agents. Three phosphoinositide-3-kinase-related kinases (PIKKs), ataxia telangiectasia mutated (ATM), ATM- and Rad3-related (ATR) and DNA-dependent protein kinase (DNA-PK), can orchestrate activation of the DDR via phosphorylation of multiple target proteins involved in cell cycle regulation, apoptosis and DNA repair. ATM and DNA-PK are primarily activated following formation of double-strand breaks (DSBs) in DNA. DSBs are recognised and bound by the MRE11-RAD50-NBS1 (MRN) complex that facilitates ATM recruitment and activation. ATM subsequently activates effector molecules that include H2AX, CHK2, KAP1, SMC1 and NBS1 that mediate cell cycle arrest and DNA repair. DNA-PK is a core component of the non-homologous end-joining (NHEJ) pathway that acts throughout the cell cycle to repair DSBs [8]. DNA-PK is activated after the Ku70/Ku80 heterodimer binds DNA ends and recruits the DNA-PK catalytic subunit (DNA-PKcs) to form the DNA-PK holoenzyme that is critical for completion of NHEJ [9]. In conjunction with ATR, DNA-PK also plays a role in maintaining genome stability following replication stress [10]. ATR is activated by a number of different lesions that result in persistent single-stranded DNA (ssDNA) that is first coated by replication protein A (RPA) before recruitment of ATR via its binding partner, ATR-interacting protein (ATRIP) [11]. The recruitment of DNA topoisomerase 2-binding protein 1 (TOPBP1) contributes to activation of ATR which subsequently orchestrates activation of cell cycle checkpoints via phosphorylation of CHK1 [12,13].

Several species of herpesvirus are known to interact with the DDR during their lifecycles. These interactions can involve the selective activation and deactivation of DDR pathways and the use of specific DDR proteins in the replication of viral genomes. Since the DDR has evolved to protect the genome from the accumulation of harmful mutations, the modulation of these pathways by human tumour viruses may contribute to the malignant transformation of host cells. KSHV has previously been shown to activate the DDR during *de novo* infection of primary endothelial cells and this plays a role in establishing latency [14]. More recently, it has been demonstrated that lytic replication of KSHV in B cells results in increased phosphorylation of H2AX, a sensitive marker for the presence of DNA damage [15,16]. It has also been demonstrated that expression of immediate-early lytic protein ORF57 alone can cause DNA damage through sequestration of the hTREX complex leading to R-loop formation and ultimately DSBs [16]. Here a more detailed assessment of DDR pathways activated during lytic replication of KSHV is presented and the effect of inhibition of the major DDR kinases on replication of viral DNA is examined. In addition, changes in the localisation of several DDR proteins in cells containing lytic virus is assessed.

## 2. Materials and Methods

### 2.1. Cell Culture

TRE-BCBL-1-RTA cells (generously provided by Jae Jung, USC, Los Angeles, CA, USA) and BCBL-1 cells were cultured in RPMI (Sigma, St. Louis, MO, USA) supplemented with 10% fetal bovine serum (FBS) (Sigma) and 1% penicillin-streptomycin (Gibco, Grand Island, NY, USA). TRE-BCBL-1-RTA cells were also cultured in the presence of 100 μg/mL of Hygromycin B (Roche, Burgess Hill, UK). The endothelial cell line, EA.hy926 (purchased from ATCC, Manassas, VA, USA), was grown in DMEM (Sigma) supplemented with 10% FBS and 1% penicillin-streptomycin. EA.hy926-RTA cells, transduced with an Inducer 20 lentivirus [17] which expresses RTA under the control of the tetracycline promoter, were cultured in the presence of 250 μg/mL of G418 (Sigma). rKSHV-EA.hy926-RTA cells, which contain the RTA expression construct and are also infected with recombinant rKSHV.219 virus [18], were cultured in the presence of 250 μg/mL of G418 and 1 μg/mL Puromycin (Sigma).

### 2.2. Induction of Lytic Reactivation in KSHV-Infected Cell Lines

To assess DDR activation in response to KSHV lytic reactivation, TRE-BCBL-1-RTA cells and rKSHV-EA.hy926-RTA cells, as well as corresponding controls, were treated with 0.5 μg/mL doxycycline (Sigma) and subsequently harvested at the indicated times for western blot analysis. To generate positive controls for DDR activation, TRE-BCBL-1-RTA cells were either exposed to 6 Gy ionising radiation (IR) and harvested after 1 h or exposed to 20 Jm^−2^ ultraviolet light (UV) and harvested after 6 h. To inhibit viral DNA synthesis, TRE-BCBL-1-RTA cells were first treated with 100 μM ganciclovir (Cayman Chemical, Ann Arbor, MI, USA) for 2 h prior to the addition of 0.5 μg/mL doxycycline.

### 2.3. Inhibition of DDR Kinases during Lytic Replication

The ATR inhibitor VE-821, ATM inhibitor KU55933 and DNA-PK inhibitor NU7441 were purchased from Tocris Bioscience (Bristol, UK). TRE-BCBL-1-RTA cells were treated with specified concentrations of kinase inhibitors, or equivalent DMSO control, 1 h prior to the addition of 0.5 μg/mL doxycycline. Cells were harvested after 24 and 48 h for western blot or immunofluorescence microscopy analysis while supernatants were collected and stored at 4 °C for assessment of infectious virus production.

### 2.4. Infection of EA.hy926 Cells with TRE-BCBL-1-RTA-Derived KSHV Virus

Supernatants collected from TRE-BCBL-1-RTA cells were added to EA.hy926 cells cultured in 6-well plates or on coverslips in 24-well plates. Cells were centrifuged (330× *g*, 20 min, ambient temperature) and following 4 h incubation at 37 °C, medium containing virus was removed and replaced with supplemented DMEM medium. After 48 h, cells were harvested for western blot analysis or fixed for immunofluorescence microscopy analysis of LANA expression.

### 2.5. Cell Cycle Analysis

Propidium iodide (PI) staining was used to determine cell cycle distribution in TRE-BCBL-1-RTA cells. Cells were treated with 100 μM ganciclovir for 1 h followed by DDR kinase inhibitors or DMSO for 1 h prior to addition of 0.5 μg/mL doxycycline. TRE-BCBL-1-RTA cells treated with ganciclovir and DMSO but not doxycycline were used as un-reactivated controls. At each time point specified, cells were washed in phosphate buffered saline (PBS) and fixed in cold 70% ethanol before storage at −20 °C. Following two PBS washes, cells were treated with PBS containing RNase A (20 μg/mL) (Sigma) and PI (10 μg/mL) (Sigma) before being analysed using an Accuri C6 flow cytometer (BD, Oxford, UK).

### 2.6. Immunofluorescence Microscopy (IF)

Following treatment with 0.5 μg/mL doxycycline for 24 h, TRE-BCBL-1-RTA cells were washed in PBS and allowed to adhere on poly-l-lysine coated glass slides (Sigma). EA.hy926-RTA cells, cultured on coverslips, were infected with KSHV derived from TRE-BCBL-1-RTA cells as described above. After 48 h, EA.hy926-RTA cells were treated with 0.5 μg/mL doxycycline for 24 h. Both cell lines were fixed with 4% paraformaldehyde and permeabilised with 0.5% Triton X-100. Cells were then blocked in 10% heat-inactivated goat serum (HINGS) before addition of primary antibodies diluted in 10% HINGS for 1 h. Cells were washed 3 times in PBS before addition of appropriate secondary antibodies diluted in 10% HINGS for 1 h. Following three PBS washes, cells were stained with DAPI nucleic acid stain (Life Technologies, Grand Island, NY, USA) and cover slides were applied using ProLong Gold antifade reagent (Life Technologies). Images were taken on a Leica DM6000B epifluorescence microscope with Leica Application Suite Advanced Fluorescence software (LAS AF) (Leica, Milton Keynes, UK).

For BrdU staining, TRE-BCBL-1-RTA cells were treated with 10 μl BrdU (Sigma) for 1 h prior to adherence on poly-l-lysine coated glass slides. Cells were then fixed in 70% ethanol for 30 min followed by treatment with 0.07 M NaOH for 2 min prior to addition of FITC-conjugated BrdU antibody for 30 min. Visualisation of viral proteins was then carried out as above.

The following primary antibodies were used for immunofluorescence microscopy: anti-γH2AX (S139) (05-636, Merck Millipore, Billerica, MA, USA), anti-RPA32 (NA19L, Calbiochem, Billerica, MA, USA), anti-MRE11 (GTX70212, Genetex, Irvine, USA), anti-53BP1 (ab36823, Abcam, Cambridge, UK), anti-K8α (SAB5300152, Sigma), anti-ORF6 (Provided by Gary Hayward) anti-ORF59 (in house), anti-LANA (NCL-HHV8-LNA, Novacastra, Newcastle, UK), anti-BrdU (347583, BD).

### 2.7. Western Blot Analysis

Lysates were prepared by re-suspending cell pellets in 8 M urea, 50 mM Tris HCl (pH 7.4) buffer containing 1% β-mercaptoethanol. Samples were then sonicated, centrifuged (16,300× *g*, 20 min, 4 °C) and stored at −80 °C. Protein determination was carried using Bradford reagent (Bio-Rad, Hercules, CA, USA) and protein standards of known concentration. Lysates were then subjected to Sodium Dodecyl Sulphate-Polyacrylamide Gel Electrophoresis (SDS-PAGE).

The following primary antibodies were used for Western blotting: anti-DNA-PKcs (04-1024, Merck Millipore), anti-phospho-DNA-PKcs (S2056) (ab124918, Abcam), anti-ATM (2873, Cell Signaling, Danvers, MA, USA), anti-phospho-ATM (S1981) (AF1655, R&D Systems, Minneapolis, MN, USA), anti-SMC1 (A300-055A, Bethyl, Montgomery, AL, USA) anti-phospho-SMC1 (S966) (A300-050A, Bethyl), anti-KAP1 (A300-274A, Bethyl), anti-phospho-KAP1 (S824) (A300-767A, Bethyl), anti-NBS1 (ab23996, Abcam), anti-phospho-NBS1 (S343) (ab47272, Abcam), anti-CHK1 (sc-8408, Santa Cruz, Dallas, TX, USA), anti-phospho-CHK1 (S345) (2341, Cell Signaling), anti-phospho-CHK1 (S317) (2344, Cell signaling), anti-CHK2 (2662, Cell Signaling), anti-phospho-CHK2 (T68) (2661, Cell Signaling), anti-RPA32 (NA19L, Calbiochem), anti-phospho-RPA32 (S4/S8) (A300-245A, Bethyl), anti-phospho-RPA32 (S33) (ab87278, Abcam), anti-H2AX (7631, Cell Signaling), anti-γH2AX (S139) (05-636, Merck Millipore), anti-β-Actin (A2228, Sigma), anti-LANA (NCL-HHV8-LNA, Novacastra), anti-RTA (in house), anti-ORF57 (AP15004a, Abgent, San Diego, CA, USA), anti-K8.1A (in house).

The following HRP conjugated secondary antibodies were also used: Polyclonal Goat Anti-Mouse (Dako Laboratories, Glostrup, Denmark), polyclonal Swine Anti-Rabbit (Dako Laboratories) and polyclonal Rabbit Anti-Goat (Dako Laboratories). Proteins were visualised using a Fusion SL chemiluminescence imaging system (Vilber Lourmat, Marne-la-Vallée, France).

## 3. Results

### 3.1. Lytic Reactivation of KSHV in B Cells Activates DDR Pathways

**Figure 1:**
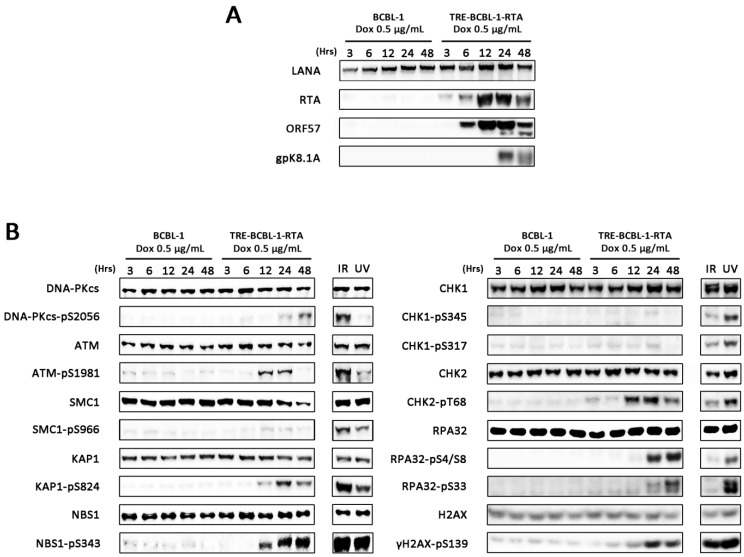
Activation of the DDR during lytic replication of KSHV in TRE-BCBL-1-RTA cells. (**A**) Expression of immediate-early lytic proteins RTA and ORF57 and late lytic glycoprotein K8.1A in TRE-BCBL-1-RTA cells following addition of 0.5 μg/mL doxycycline; (**B**) Phosphorylation of DDR proteins in TRE-BCBL-1-RTA cells following addition of 0.5 μg/mL doxycycline. TRE-BCBL-1-RTA cells treated with ionising radiation (IR) and ultraviolet light (UV) represent positive controls for DDR activation.

Analysis of DDR pathway activation during KSHV lytic replication was initially performed on a derivative of the BCBL-1 PEL line that is latently infected with KSHV. These cells, called TRE-BCBL-1-RTA, have been modified to include the ORF50/RTA gene under the control of a tetracycline promoter [19]. Addition of doxycycline to TRE-BCBL-1-RTA cells reactivates KSHV into the lytic cycle resulting in expression of immediate-early lytic proteins such as ORF57 followed by late proteins such as envelope glycoprotein K8.1A and eventually release of infectious virions (Figure 1A). Using this cell line and control BCBL-1 cells lacking the RTA-expression construct, levels of phosphorylated DDR proteins were assessed by western blotting at 5 time points following addition of doxycycline. TRE-BCBL1-RTA cells exposed to either IR or UV were used as positive controls for DDR activation. Addition of doxycycline to control BCBL-1 cells did not result in phosphorylation of any of the DDR proteins examined. While addition of doxycycline to TRE-BCBL-1-RTA cells resulted in minimal phosphorylation of SMC1 and CHK1 compared with IR and UV treated controls, after 12 h there was a notable increase in levels of phosphorylated ATM, DNA-PKcs, CHK2, KAP1, NBS1, RPA32 and H2AX indicating widespread activation of the DDR during the KSHV lytic cycle (Figure 1B).

### 3.2. Amplification of Viral DNA and Late Viral Gene Expression Is Not Required for DDR Activation

**Figure 2:**
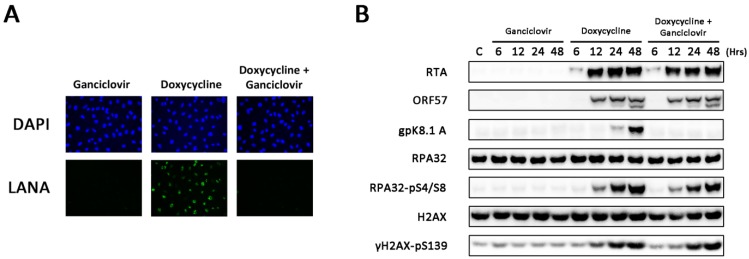
DDR activation following inhibition of viral DNA synthesis and late viral gene expression. (**A**) Immunofluorescence microscopy analysis of LANA expression in EA.hy926 cells following infection with supernatants obtained from TRE-BCBL-1-RTA cells collected 48 h after treatment with 100 μM ganciclovir alone, 0.5 μg/mL doxycycline alone or both compounds in combination; (**B**) Phosphorylation of RPA32 and H2AX in TRE-BCBL-1-RTA cells treated with 100 μM ganciclovir alone, 0.5 μg/mL doxycycline alone or both compounds in combination.

To determine whether amplification of viral DNA or late viral gene expression is required for DDR activation in TRE-BCBL-1-RTA cells, the antiviral compound ganciclovir was employed. Ganciclovir inhibits viral DNA synthesis without affecting the expression of early lytic proteins or replication of host DNA [20]. TRE-BCBL-1-RTA cells were treated with and without 100 μM ganciclovir prior to the addition of doxycycline. Cells were also treated with ganciclovir alone to ensure that this compound in isolation did not activate the DDR. To ensure that ganciclovir was efficiently inhibiting production of infectious virus, TRE-BCBL-1-RTA supernatants from each condition were collected after 48 h and used to infect EA.hy926 cells. Expression of LANA was assessed by immunofluorescence microscopy as a marker of KSHV infection. Minimal LANA expression was observed in EA.hy926 cells infected with supernatants from TRE-BCBL-1-RTA cells treated with doxycycline and ganciclovir or ganciclovir alone (Figure 2A). Western blots confirmed that the cells treated with ganciclovir and doxycycline expressed the immediate-early lytic proteins RTA and ORF57 but not the late envelope glycoprotein K8.1A (Figure 2B). Levels of phosphorylated H2AX and RPA32 were unchanged in the doxycycline-treated cells despite the presence of ganciclovir suggesting that DDR activation is mediated by early viral gene expression and does not require amplification of viral DNA or expression of late lytic proteins. 

### 3.3. Inhibition of DDR Kinases Results in Alterations in Production of Infectious Virus

**Figure 3:**
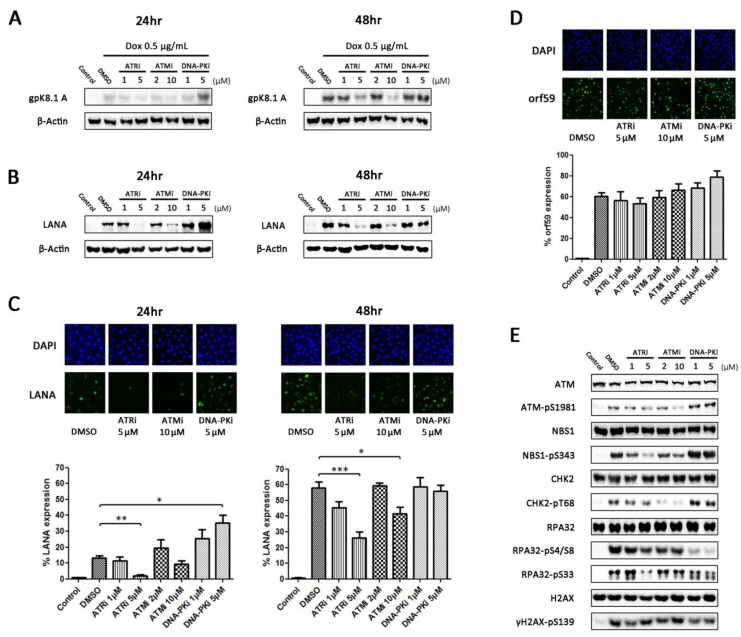
Effect of DDR inhibition on production of infectious virus. (**A**) Expression of late lytic glycoprotein K8.1A in TRE-BCBL-1-RTA cells 24 and 48 h following treatment with DDR kinase inhibitors and doxycycline; (**B**) Expression of LANA in EA.hy926 cells following infection with supernatants collected from TRE-BCBL-1-RTA cells 24 and 48 h after treatment with DDR kinase inhibitors and doxycycline; (**C**) Immunofluorescence microscopy analysis of the percentage of EA.hy926 cells expressing LANA following infection with supernatants collected from TRE-BCBL-1-RTA cells 24 and 48 h after treatment with DDR kinase inhibitors and doxycycline. Each column represents the mean of three independent experiments while the error bars represent the standard error of the mean (SEM). A minimum of 500 cells were analysed for each repetition. * *p* < 0.05; ** *p* < 0.01; *** *p* < 0.001 (statistical analyses were performed using a two-tailed and unpaired Student’s *t*-test); (**D**) Percentage of TRE-BCBL-1-RTA cells expressing early lytic protein ORF59 24 h following treatment with DDR kinase inhibitors and doxycycline. Each column represents the mean of three independent experiments while the error bars represent the standard error of the mean (SEM). A minimum of 500 cells were analysed for each repetition; (**E**) Levels of phosphorylated DDR proteins in TRE-BCBL-1-RTA cells 24 h following treatment with DDR kinase inhibitors and doxycycline.

To determine what effect inhibition of DDR kinases has on production of infectious virus, TRE-BCBL-1-RTA cells were treated with two concentrations of ATR, ATM and DNA-PK inhibitors or an equivalent concentration of DMSO 1 h prior to the addition of 0.5 μg/mL doxycycline. After 24 and 48 h, expression of late lytic glycoprotein K8.1A was initially assessed as an indicator of completed viral replication (Figure 3A). At 24 h, expression of K8.1A was relatively low compared to 48 h but was clearly elevated following inhibition of DNA-PK. After 48 h, expression was similar between the DMSO-treated and DNA-PK inhibitor-treated cells but clearly reduced in the cells treated with the higher concentrations of ATR and ATM inhibitors.

To ensure that levels of K8.1A expression were representative of the amount of infectious virus produced from TRE-BCBL1-RTA cells, the medium supernatants were collected from the cells after 24 and 48 h and used to infect the EA.hy926 endothelial cell line. After 48 h, western blotting was used to assess expression of LANA as a marker of KSHV infection (Figure 3B). The expression levels of LANA in the EA.hy926 cells correlated well with the levels of K8.1A expression in TRE-BCBL-1-RTA cells at both 24 and 48 h. To quantify further the level of virus contained in the supernatants, the proportion of EA.hy926 cells expressing LANA was calculated using immunofluorescence microscopy (Figure 3C). In the EA.hy926 cells treated with the 24 h supernatants there was a significant increase in the proportion of cells expressing LANA following infection with the 5 μM DNA-PKi supernatants and a significant decrease following infection with the 5 μM ATRi supernatants. In the EA.hy926 cells treated with the 48 h supernatants there was a significant decrease in the proportion of cells expressing LANA following infection with the 5 μM ATRi and 10 μM ATMi supernatants.

Since not all TRE-BCBL-1-RTA cells contain lytically replicating virus following treatment with doxycycline, it is possible that the differences observed in infectious virus production are due to changes in the percentages of cells containing lytic virus rather than changes in the amount of virus produced from individual cells. To quantify the percentage of cells containing lytic virus following treatment with DDR inhibitors and doxycycline, expression of delayed-early lytic protein ORF59 was assessed using immunofluorescence microscopy (Figure 3D). Although the percentage of cells expressing ORF59 appears elevated following the addition of DNA-PK inhibitor there were no significant differences between any of the conditions. This suggests that the changes in production of infectious virus are not because of differing numbers of cells containing lytic virus but because of changes in the efficiency of viral replication.

Several proteins involved in DDR signalling can be phosphorylated by more than one of the three key DDR kinases. To examine which kinase was responsible for the phosphorylation events observed during lytic replication of KSHV, changes in phosphorylation level of several DDR proteins in TRE-BCBL-1-RTA cells was assessed 24 h following addition of DDR inhibitors and doxycycline (Figure 3E). Inhibition of DNA-PK markedly reduced phosphorylation of RPA32 at S4/S8 and moderately reduced phosphorylation of H2AX at S139 and RPA32 at S33. DNA-PK inhibition also led to an increase in ATM, NBS1 and CHK2 phosphorylation. Inhibition of ATM strongly reduced CHK2 phosphorylation as expected but only partially reduced phosphorylation of NBS1. Inhibition of ATR reduced phosphorylation of RPA32 at S33 and reduced NBS1 phosphorylation to a greater extent than the ATM inhibitor. This indicates that DNA-PK and ATM are activating downstream targets during lytic replication but also suggests active ATR despite the lack of CHK1 phosphorylation.

### 3.4. CHK1 Activation is Inhibited at Later Times during Lytic Replication

**Figure 4:**
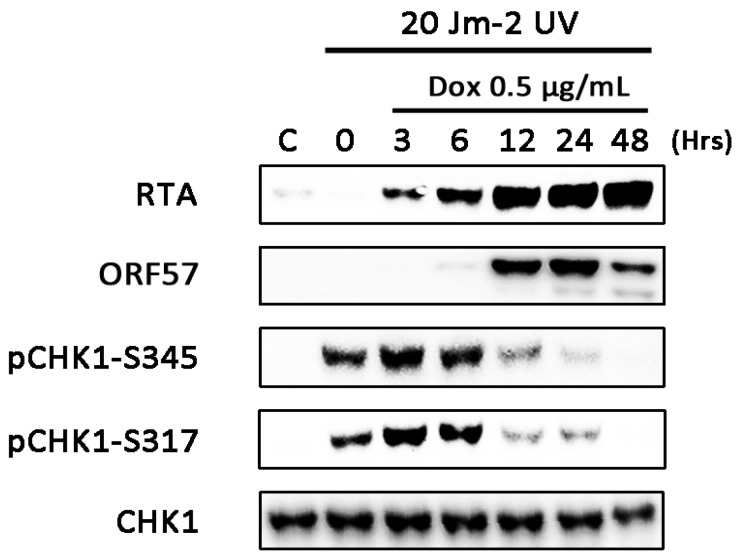
Inhibition of CHK1 signalling at late times during KSHV lytic replication. Levels of phosphorylated CHK1 (S317 and S345) following UV treatment of TRE-BCBL-1-RTA cells treated with doxycycline for varying lengths of time. At each time point following the addition of doxycycline, cells were treated with 20 Jm^−^^2^ UV and levels of phosphorylated CHK1 were assessed by western blot.

While there is evidence of ATR activation during lytic replication, substantial CHK1 phosphorylation is not observed (Figure 1B). To determine whether ATR-CHK1 signalling is functional during lytic replication, TRE-BCBL-1-RTA cells were exposed to 20 Jm^−2^ UV at several time points following initiation of lytic replication (Figure 4). Expression of RTA and ORF57 were used to confirm lytic reactivation of KSHV. Exposure to UV at 3 and 6 h post-doxycycline treatment led to phosphorylation of CHK1 at S345 and S317 which was comparable to the un-reactivated control. However, when TRE-BCBL-1-RTA cells were treated with UV at 12, 24 and 48 h following addition of doxycycline, levels of phosphorylated CHK1 were noticeably lower than in the un-reactivated control. This suggests that at later times in the lytic cycle, when phosphorylation of other DDR substrates occurs, activation of CHK1 is specifically inhibited by the virus.

### 3.5. DDR Activation Does Not Result in a G1 Block Following Lytic Reactivation

**Figure 5:**
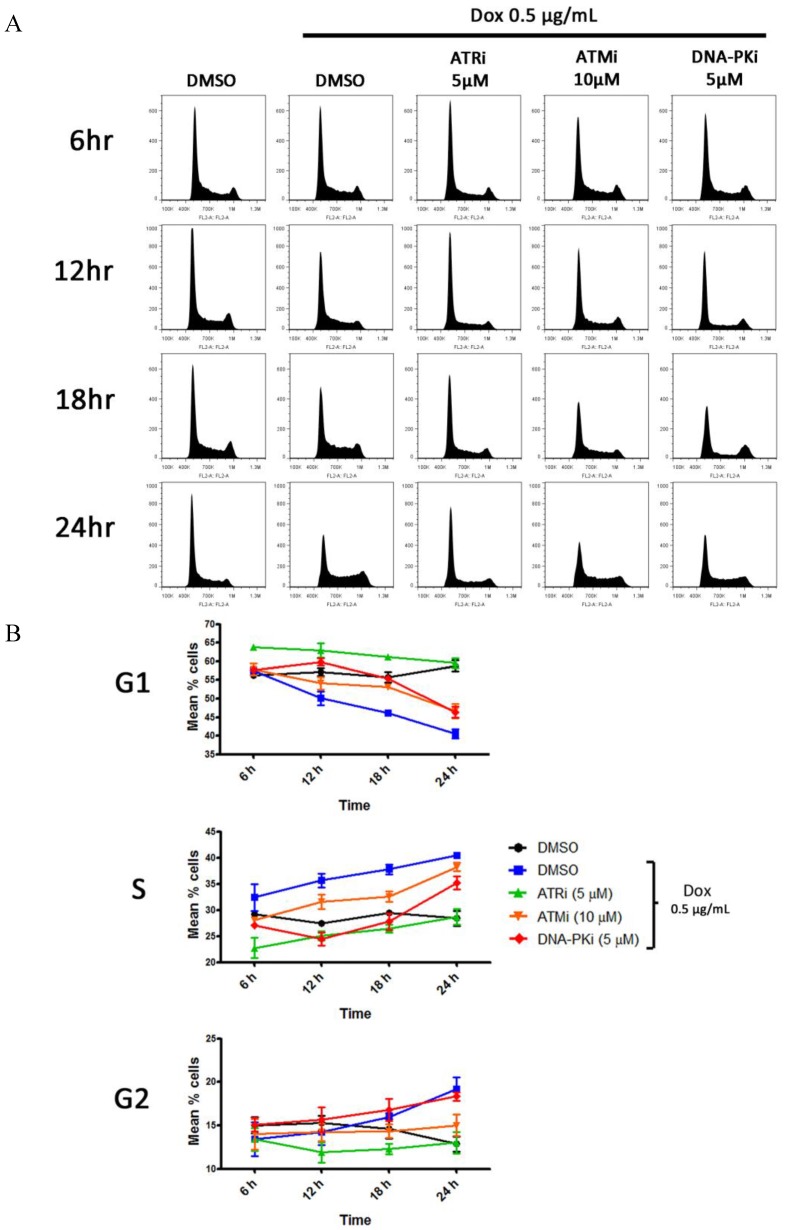
Cell cycle changes during lytic replication of KSHV. (**A**) Representative cell cycle profiles following PI staining of TRE-BCBL-1-RTA cells after addition of doxycycline in the presence and absence of DDR kinase inhibitors; (**B**) Percentage of TRE-BCBL-1-RTA cells in different phases of the cell cycle at 4 different time points following addition of doxycycline in the presence and absence of DDR kinase inhibitors. Each data point represents the mean of three independent experiments while the error bars represent the standard error of the mean (SEM).

Since viruses are known to modulate the cell cycle to facilitate viral replication and DDR activation can vary throughout the cell cycle, an assessment of cell cycle changes during lytic replication and in the presence of DDR kinase inhibitors was undertaken (Figure 5). TRE-BCBL-1-RTA cells were treated with DMSO or the higher concentrations of DDR kinase inhibitors 1 h prior to the addition of doxycycline. Cells were harvested after 6, 12, 18 and 24 h and subjected to PI staining. Because production of viral DNA by herpesviruses has been shown to interfere with PI staining [21], all TRE-BCBL-1-RTA cells were also treated with ganciclovir 1 h prior to the addition of DDR inhibitors or DMSO. At the 6 and 12 h time points the experiment was also conducted without ganciclovir to confirm that this compound alone has no significant effects on the cell cycle distribution following addition of doxycycline (data not shown). Cell counts also confirmed that ganciclovir alone had no significant effect on the growth rate of TRE-BCBL-1-RTA cells over 24 h (data not shown).

The addition of doxycycline to cells treated with DMSO resulted in a decrease in the proportion of cells in G1 and an increase in the proportion in S-phase during the time course compared with the un-reactivated control. By 24 h there was also a notable increase in the proportion of cells in G2 following doxycycline treatment. Addition of the ATR inhibitor prior to doxycycline increased the proportion of cells in G1 and decreased the proportion in S and G2 compared with the cells treated with doxycycline and DMSO. By 24 h, the cell cycle profile in the presence of the ATR inhibitor and doxycycline was almost identical to the un-reactivated control. While treatment with the ATM and DNA-PK inhibitors also initially increased the proportion of cells in G1, by 24 h the difference between these conditions and the cells treated with doxycycline and DMSO was minimal. It appears then that DDR activation following lytic activation does not cause a G1 cell cycle block and instead cells are able to proceed to S and G2 phases. Addition of the ATR inhibitor, however, appears to inhibit the cell cycle changes normally induced during the lytic cycle.

### 3.6. RPA32 and MRE11 Localise to Sites of Viral Replication

**Figure 6:**
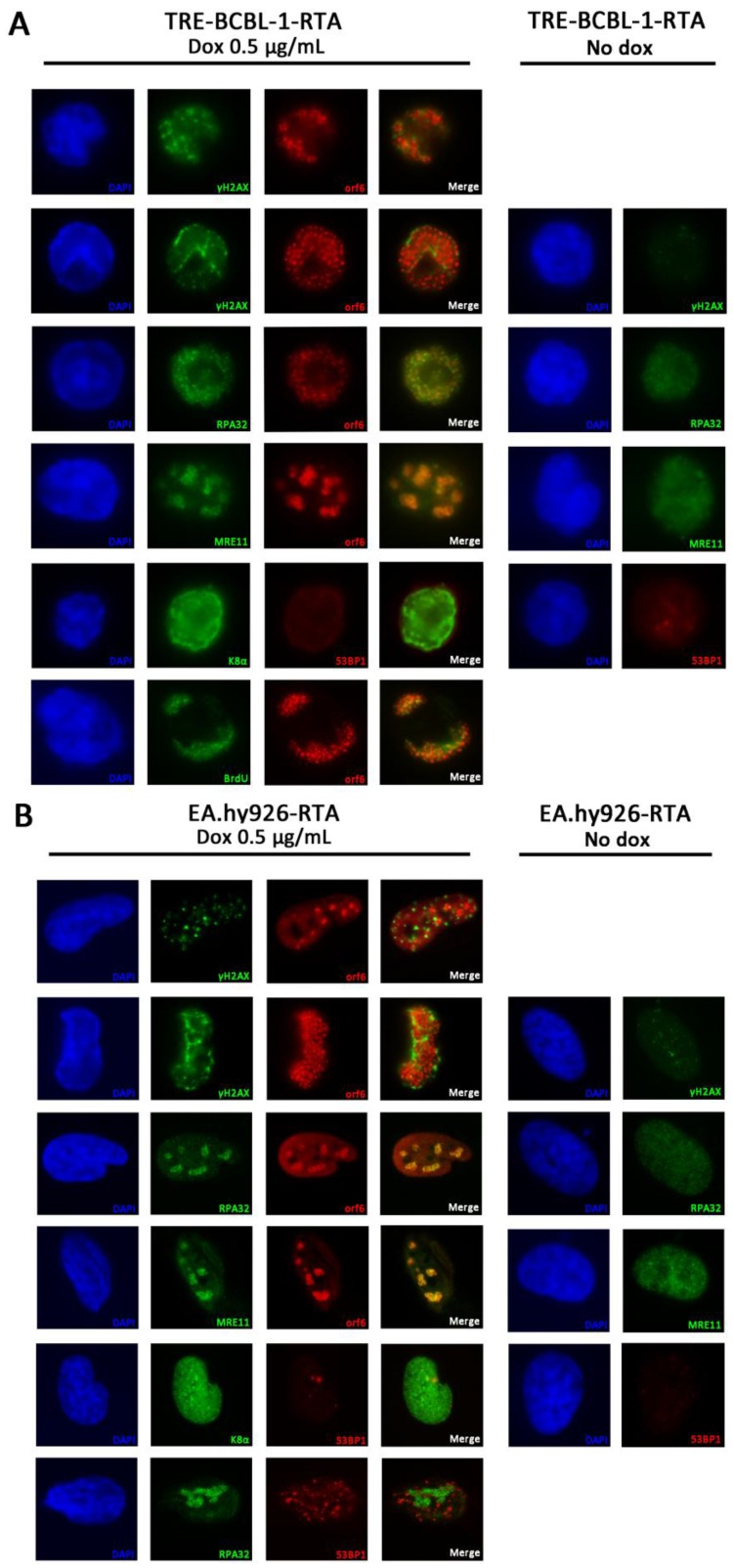
Localisation of DDR proteins during lytic replication of KSHV. (**A**) Localisation of γH2AX, RPA32, MRE11 and 53BP1 in TRE-BCBL-1-RTA cells 24 h following addition of doxycycline and in cells without doxycycline treatment. KSHV lytic proteins K8α and ORF6 were used as markers of lytic reactivation. In the bottom panels, BrdU was used to confirm that ORF6 localises to sites of viral replication; (**B**) Localisation of γH2AX, RPA32, MRE11 and 53BP1 in KSHV-infected EA.hy926-RTA cells 24 h following addition of doxycycline and in cells without doxycycline treatment. KSHV lytic proteins K8α and ORF6 were used as markers of lytic reactivation. In the bottom panels, RPA32 was used as a surrogate marker for sites of viral replication. DAPI staining is only shown in the left hand set of panels in each case for clarity.

Several DDR proteins are known to form discrete foci at sites of DNA damage [22]. In addition, several herpesviruses are known to recruit DDR proteins to sites of viral replication where they may be involved in viral DNA synthesis [23,24,25]. An assessment of the localisation of several DDR proteins was undertaken 24 h after induction of KSHV lytic replication (Figure 6). As well as using TRE-BCBL-1-RTA cells (Figure 6A), EA.hy926-RTA cells were examined as their larger size and flat morphology allowed for clearer visualisation of protein localization (Figure 6B). Expression of lytic proteins ORF6 and K8α were used as markers of KSHV lytic replication. The delayed-early lytic protein ORF6 has been shown previously to localise to sites of viral DNA replication in BCBL-1 cells [26]. BrdU staining was used to confirm that ORF6-positive domains were sites of viral DNA synthesis (Figure 6A).

Phosphorylated H2AX is a sensitive marker for the presence of DNA damage and γH2AX foci were consistently observed in TRE-BCBL-1-RTA and EA.hy926 cells positive for ORF6 expression. When ORF6 was localised to discrete replication bodies in EA.hy926-RTA cells, γH2AX foci typically formed outside of these bodies in cellular DNA while in TRE-BCBL-1-RTA cells γH2AX foci appear to form on the margins of these replication compartments. In cells with larger replication bodies, γH2AX staining could be seen on the periphery of replication domains as cellular DNA is marginalised to the edge of the nucleus. RPA32, which binds to single-stranded DNA, also forms foci following DNA damage. In this case, RPA32 was consistently observed localised in the same areas of the nucleus as ORF6 in both TRE-BCBL-1-RTA and EA.hy926-RTA cells, suggesting an association with viral replication domains. In addition, MRE11, part of the MRN complex that localises to DSBs, was also found to localise to viral replication compartments in both cell lines.

The localisation of 53BP1 was also examined as this protein is known to form foci at sites of DSBs [27]. Despite the activation of ATM and DNA-PK in TRE-BCBL-1-RTA cells, consistent formation of 53BP1 foci was not observed in cells positive for K8α after 24 h. In EA.hy926-RTA cells, where it is possible to accurately quantify the number of foci present, less than 30% of cells positive for K8α contained more than ten 53BP1 foci. As K8α did not appear to localise to replication domains in EA.hy926-RTA cells, and since RPA32 was consistently observed in association with ORF6, RPA32 was used as surrogate marker for KSHV replication domains when staining for 53BP1. When 53BP1 did form foci there was no evidence of localisation to KSHV replication domains.

### 3.7. Lytic Reactivation of KSHV in an Endothelial Cell Line Activates the DDR

**Figure 7:**
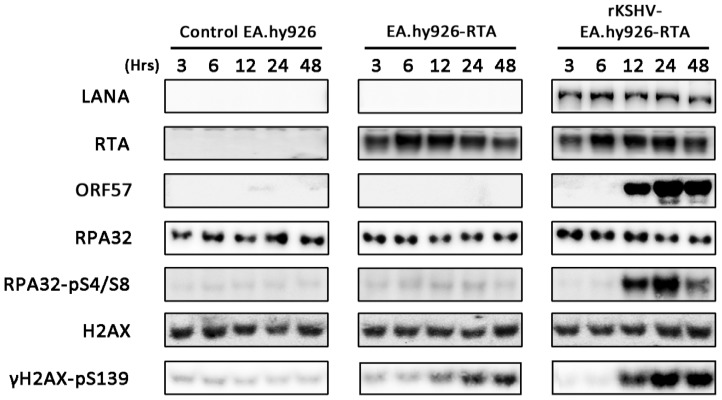
Activation of the DNA damage response during lytic replication of KSHV in EA.hy926 cells and following RTA expression alone. Levels of phosphorylated H2AX and RPA32 following addition of doxycyline to control EA.hy926 cells, EA.hy926 cells containing an ORF50 expression construct (EA.hy926-RTA) and KSHV-infected EA.hy926 cells containing an ORF50 expression construct (rKSHV-EA.hy926-RTA). Expression of LANA and ORF57 confirm presence of KSHV and lytic reactivation of the virus respectively.

Since KSHV targets both B cells and endothelial cells *in vivo*, activation of the DDR during KSHV lytic replication was also assessed in the EA.hy926 endothelial cell line (Figure 7). As with the TRE-BCBL-1-RTA cells, DDR activation was compared between uninfected EA.hy926 cells and KSHV-infected EA.hy926 cells containing an RTA expression construct (termed rKSHV-EA.hy926-RTA) following treatment with doxycycline. In addition, uninfected EA.hy926 cells containing the RTA expression construct (termed EA.hy926-RTA) were also used to assess the effect of RTA expression alone on DDR activation. All three cell lines were treated with doxycycline and levels of phosphorylated H2AX and RPA32 were used to assess DDR activation at the same times examined in TRE-BCBL-1-RTA cells. Expression of LANA and ORF57 were used to confirm KSHV infection and lytic reactivation in the rKSHV-EA.hy926-RTA cell line. Induction of lytic replication in rKSHV-EA.hy926-RTA cells led to DDR activation with increased phosphorylation of RPA32 and H2AX. Expression of RTA alone in EA.hy926-RTA cells also increased phosphorylation of H2AX but to a limited extent compared to cells containing KSHV.

## 4. Discussion

It has been demonstrated here that during lytic replication of KSHV, the ATM and DNA-PK kinases are activated and phosphorylate several downstream targets. The timing of some of these phosphorylation events suggests that immediate-early or delayed-early lytic gene expression is responsible for DDR activation. The use of ganciclovir to inhibit viral DNA synthesis and late viral gene expression confirms that these stages of the lytic cycle are not required for the observed activation of the DDR. In accordance with this, it was recently reported that expression of KSHV immediate early lytic protein ORF57 can cause DNA damage and phosphorylation of H2AX when expressed in isolation [16].

It has also been shown here that inhibition of ATR and ATM results in reduced production of infectious virus following induction of lytic replication in B cells. Inhibition of ATM signalling has also been shown to reduce replication of the related gamma herpesvirus Epstein-Barr virus (EBV) in several studies [28,29,30]. Although the reduction in viral replication following ATM inhibition was moderate, DNA-PK inhibition led to increased phosphorylation of ATM and downstream substrates and increased production of infectious virus after 24 h. This observation also suggests that activation of the ATM-CHK2 arm of the DDR can promote efficient KSHV replication in B cells. The abrogation of ATM signalling was not completely efficient even at the higher concentration of ATM inhibitor. While increased concentrations of inhibitor were avoided due to concerns about toxicity and off-target effects, it would be interesting to see if a more complete knockdown of ATM could result in a more dramatic inhibitory effect on viral replication. This could potentially be achieved using Crispr/Cas in further investigations.

The inhibition of ATR resulted in the largest reduction in virus production although the lack of CHK1 phosphorylation prompts the question of whether this kinase is activated during the lytic cycle. While ATR inhibition reduced phosphorylation of NBS1 and RPA32, both of which are known ATR substrates [31,32], it is possible that cell cycle changes induced by the ATR inhibitor could contribute to a reduction in phosphorylation of these proteins by ATM and DNA-PK as well as the observed decline in virus production, assuming that progression into S-phase is advantageous (Figure 5). Whereas phosphorylation of CHK1 at serines 317 and 345 are typical markers for ATR activation in response to replication stress [33], the ATR-CHK1 pathway can also be activated when repair of DSBs during S and G2 phases generates RPA-coated ssDNA [34,35]. It was interesting therefore to observe that there was minimal CHK1 phosphorylation even at late times during lytic replication. In herpes simplex virus 1 (HSV-1), ATR signalling is disabled during viral replication and proteins involved in the ATR-CHK1 pathway are recruited to viral replication centres [25]. It appears that ATR-CHK1 signalling is disrupted at later times during KSHV lytic replication following treatment with UV. It is not clear at this stage whether inhibition of CHK1 is a deliberate viral strategy or merely a consequence of wider DDR deregulation during lytic replication.

It was notable that a G1 block was not observed during the first 24 h following lytic replication considering that this has been reported in several studies of herpesviruses including KSHV [21,36,37,38,39]. KSHV lytic proteins RTA and K8α have both been shown to induce a G1 block when expressed in isolation [37,38,39]. However, KSHV also encodes a D-cyclin homolog, known as v-cyclin, that has been reported to induce cell cycle progression via inactivation of p27^KIP1^ [40]. v-cyclin is expressed throughout the viral lifecycle but expression is increased following lytic reactivation [41]. It has also been shown to activate the DDR in endothelial cells by promoting deregulated S-phase entry [42]. Considering the apparent opposing effects of these viral proteins on cell cycle progression, and the large number of genes expressed during the lytic cycle, it is clear that single gene expression studies could lead to contradictory finding in this area. While it has been postulated that replication in G1 is ideal during the lytic cycle as the virus avoids competition for DNA replication resources [37], there is also evidence that S-phase can provide KSHV with a favourable environment for viral replication [43]. The data presented here suggest that, despite the presence of DNA damage, cells containing lytic virus can still progress into S-phase and that when a G1 block is induced by inhibition of ATR it has a negative effect on the efficient production of viral DNA.

The activation of ATM and DNA-PK suggests the formation of DSBs during KSHV replication. Increased phosphorylation of H2AX and the presence of γH2AX foci have previously been used to confirm the presence of DSBs in B cells following induction of the lytic cycle [15,16]. The formation of γH2AX foci was also observed during this study but since γH2AX foci can also form at single-stranded DNA breaks and stalled replication forks [44], the presence of 53BP1 foci which form rapidly at sites of DSBs was also examined. Curiously, we did not observe consistent formation of 53BP1 foci in cells positive for lytic virus. Although 53BP1 localisation has been implicated in ATM activation it is not strictly required following high levels of DNA damage [45]. In EBV, 53BP1 has been shown to be important for viral replication [46] while in human cytomegalovirus (HCMV), overall 53BP1 levels decrease during infection [24]. In HSV-1, degradation of the E3 ubiquitin ligases RNF8 and RNF168 inhibits localisation of 53BP1 to incoming viral genomes [47]. It will be interesting to assess further whether expression and localisation of this protein is deregulated during the KSHV lifecycle.

RPA32 and MRE11 can also form foci following DNA damage and both proteins were consistently observed localised to areas of viral replication; this was particularly evident in EA.hy926 cells where ORF6 staining can be seen as discrete bodies that occupy a smaller proportion of the nucleus than in B cells. Both RPA32 and MRE11 have been shown to localise to EBV replication compartments where they are loaded onto newly synthesised viral DNA along with HR factors Rad51 and Rad52 [23]. Further work will be required to determine if any other DDR factors are recruited to KSHV replication compartments and what role they play in viral replication.

Overall, it appears that lytic reactivation of KSHV leads to selective activation of the DDR and this plays a positive role in the viral lifecycle. In addition, the localisation of DDR proteins to areas of viral replication suggests that individual DDR proteins may contribute to viral DNA synthesis, as is the case with other herpesviruses. Although the data presented here do not contradict the demonstration that expression of ORF57 can elicit a DDR, our observations using an endothelial cell line suggest that RTA alone can also modestly increase phosphorylation of H2AX. It is therefore possible that expression of several early lytic proteins could be responsible for DDR activation which then contributes to efficient production of viral progeny.

## References

[1] Chang Y., Cesarman E., Pessin M.S., Lee F., Culpepper J., Knowles D.M., Moore P.S. (1994). Identification of herpesvirus-like DNA sequences in aids-associated kaposi’s sarcoma. Science.

[2] Cesarman E., Chang Y., Moore P.S., Said J.W., Knowles D.M. (1995). Kaposi’s sarcoma-associated herpesvirus-like DNA sequences in aids-related body-cavity-based lymphomas. N. Engl. J. Med..

[3] Soulier J., Grollet L., Oksenhendler E., Cacoub P., Cazals-Hatem D., Babinet P., D’Agay M.F., Clauvel J.P., Raphael M., Degos L. (1995). Kaposi’s sarcoma-associated herpesvirus-like DNA sequences in multicentric castleman’s disease. Blood.

[4] Sun R., Lin S.F., Gradoville L., Yuan Y., Zhu F., Miller G. (1998). A viral gene that activates lytic cycle expression of kaposi’s sarcoma-associated herpesvirus. Proc. Natl. Acad. Sci. USA.

[5] Guito J., Lukac D.M. (2015). Kshv reactivation and novel implications of protein isomerization on lytic switch control. Viruses.

[6] Martin D.F., Kuppermann B.D., Wolitz R.A., Palestine A.G., Li H., Robinson C.A. (1999). Oral ganciclovir for patients with cytomegalovirus retinitis treated with a ganciclovir implant. Roche ganciclovir study group. N. Engl. J. Med..

[7] Grundhoff A., Ganem D. (2004). Inefficient establishment of kshv latency suggests an additional role for continued lytic replication in kaposi sarcoma pathogenesis. J. Clin. Investig..

[8] Rothkamm K., Kruger I., Thompson L.H., Lobrich M. (2003). Pathways of DNA double-strand break repair during the mammalian cell cycle. Mol. Cell. Biol..

[9] Lieber M.R. (2010). The mechanism of double-strand DNA break repair by the nonhomologous DNA end-joining pathway. Annu. Rev. Biochem..

[10] Ashley A.K., Shrivastav M., Nie J., Amerin C., Troksa K., Glanzer J.G., Liu S., Opiyo S.O., Dimitrova D.D., Le P. (2014). DNA-pk phosphorylation of rpa32 ser4/ser8 regulates replication stress checkpoint activation, fork restart, homologous recombination and mitotic catastrophe. DNA Repair Amst..

[11] Zou L., Elledge S.J. (2003). Sensing DNA damage through atrip recognition of rpa-ssdna complexes. Science.

[12] Chini C.C., Chen J. (2003). Human claspin is required for replication checkpoint control. J. Biol. Chem..

[13] Kumagai A., Lee J., Yoo H.Y., Dunphy W.G. (2006). Topbp1 activates the atr-atrip complex. Cell.

[14] Singh V.V., Dutta D., Ansari M.A., Dutta S., Chandran B. (2014). Kaposi’s sarcoma-associated herpesvirus induces the ATM and H2AX DNA damage response early during de novo infection of primary endothelial cells, which play roles in latency establishment. J. Virol..

[15] Xiao Y., Chen J., Liao Q., Wu Y., Peng C., Chen X. (2013). Lytic infection of kaposi’s sarcoma-associated herpesvirus induces DNA double-strand breaks and impairs non-homologous end joining. J. Gen. Virol..

[16] Jackson B.R., Noerenberg M., Whitehouse A. (2014). A novel mechanism inducing genome instability in kaposi’s sarcoma-associated herpesvirus infected cells. PLoS Pathog..

[17] Meerbrey K.L., Hu G., Kessler J.D., Roarty K., Li M.Z., Herschkowitz J.I., Burrows A.E., Fang J.E., Ciccia A., Sun T. (2011). The pinducer lentiviral toolkit for inducible rna interference *in vitro* and *in vivo*. Proc. Natl. Acad. Sci. USA.

[18] Vieira J., O’Hearn P.M. (2004). Use of the red fluorescent protein as a marker of kaposi’s sarcoma-associated herpesvirus lytic gene expression. Virology.

[19] Nakamura H., Lu M., Gwack Y., Souvlis J., Zeichner S.L., Jung J.U. (2003). Global changes in kaposi’s sarcoma-associated virus gene expression patterns following expression of a tetracycline-inducible rta transactivator. J. Virol..

[20] Matthews T., Boehme R. (1988). Antiviral activity and mechanism of action of ganciclovir. Rev. Infect. Dis..

[21] Bresnahan W.A., Boldogh I., Thompson E.A., Albrecht T. (1996). Human cytomegalovirus inhibits cellular DNA synthesis and arrests productively infected cells in late g1. Virology.

[22] Polo S.E., Jackson S.P. (2011). Dynamics of DNA damage response proteins at DNA breaks: A focus on protein modifications. Genes Dev..

[23] Kudoh A., Iwahori S., Sato Y., Nakayama S., Isomura H., Murata T., Tsurumi T. (2009). Homologous recombinational repair factors are recruited and loaded onto the viral DNA genome in epstein-barr virus replication compartments. J. Virol..

[24] Luo M.H., Rosenke K., Czornak K., Fortunato E.A. (2007). Human cytomegalovirus disrupts both ataxia telangiectasia mutated protein (ATM)- and ATM-rad3-related kinase-mediated DNA damage responses during lytic infection. J. Virol..

[25] Mohni K.N., Dee A.R., Smith S., Schumacher A.J., Weller S.K. (2013). Efficient herpes simplex virus 1 replication requires cellular atr pathway proteins. J. Virol..

[26] Wang Y., Li H., Tang Q., Maul G.G., Yuan Y. (2008). Kaposi’s sarcoma-associated herpesvirus ori-lyt-dependent DNA replication: Involvement of host cellular factors. J. Virol..

[27] Anderson L., Henderson C., Adachi Y. (2001). Phosphorylation and rapid relocalization of 53bp1 to nuclear foci upon DNA damage. Mol. Cell. Biol..

[28] Li R., Zhu J., Xie Z., Liao G., Liu J., Chen M.R., Hu S., Woodard C., Lin J., Taverna S.D. (2011). Conserved herpesvirus kinases target the DNA damage response pathway and tip60 histone acetyltransferase to promote virus replication. Cell Host Microbe.

[29] Hagemeier S.R., Barlow E.A., Meng Q., Kenney S.C. (2012). The cellular ataxia telangiectasia-mutated kinase promotes epstein-barr virus lytic reactivation in response to multiple different types of lytic reactivation-inducing stimuli. J. Virol..

[30] Hau P.M., Deng W., Jia L., Yang J., Tsurumi T., Chiang A.K., Huen M.S., Tsao S.W. (2015). Role of atm in the formation of the replication compartment during lytic replication of epstein-barr virus in nasopharyngeal epithelial cells. J. Virol..

[31] Liu S., Bekker-Jensen S., Mailand N., Lukas C., Bartek J., Lukas J. (2006). Claspin operates downstream of TOPBP1 to direct atr signaling towards chk1 activation. Mol. Cell. Biol..

[32] Liu S., Opiyo S.O., Manthey K., Glanzer J.G., Ashley A.K., Amerin C., Troksa K., Shrivastav M., Nickoloff J.A., Oakley G.G. (2012). Distinct roles for DNA-pk, atm and atr in rpa phosphorylation and checkpoint activation in response to replication stress. Nucleic Acids Res..

[33] Cimprich K.A., Cortez D. (2008). ATR: An essential regulator of genome integrity. Nat. Rev. Mol. Cell. Biol..

[34] Jazayeri A., Falck J., Lukas C., Bartek J., Smith G.C., Lukas J., Jackson S.P. (2006). ATM- and cell cycle-dependent regulation of atr in response to DNA double-strand breaks. Nat. Cell Biol..

[35] Shibata A., Barton O., Noon A.T., Dahm K., Deckbar D., Goodarzi A.A., Lobrich M., Jeggo P.A. (2010). Role of ATM and the damage response mediator proteins 53bp1 and mdc1 in the maintenance of G_2_/M checkpoint arrest. Mol. Cell. Biol..

[36] Rodriguez A., Jung E.J., Flemington E.K. (2001). Cell cycle analysis of epstein-barr virus-infected cells following treatment with lytic cycle-inducing agents. J. Virol..

[37] Wu F.Y., Tang Q.Q., Chen H., ApRhys C., Farrell C., Chen J., Fujimuro M., Lane M.D., Hayward G.S. (2002). Lytic replication-associated protein (RAP) encoded by kaposi sarcoma-associated herpesvirus causes p21CIP-1-mediated G1 cell cycle arrest through ccaat/enhancer-binding protein-alpha. Proc. Natl. Acad. Sci. USA.

[38] Izumiya Y., Lin S.F., Ellison T.J., Levy A.M., Mayeur G.L., Izumiya C., Kung H.J. (2003). Cell cycle regulation by kaposi’s sarcoma-associated herpesvirus k-bzip: Direct interaction with cyclin-CDK2 and induction of G1 growth arrest. J. Virol..

[39] Kumar P., Wood C. (2013). Kaposi’s sarcoma-associated herpesvirus transactivator rta induces cell cycle arrest in G0/G1 phase by stabilizing and promoting nuclear localization of p27kip. J. Virol..

[40] Jarviluoma A., Koopal S., Rasanen S., Makela T.P., Ojala P.M. (2004). Kshv viral cyclin binds to p27kip1 in primary effusion lymphomas. Blood.

[41] Arias C., Weisburd B., Stern-Ginossar N., Mercier A., Madrid A.S., Bellare P., Holdorf M., Weissman J.S., Ganem D. (2014). Kshv 2.0: A comprehensive annotation of the kaposi’s sarcoma-associated herpesvirus genome using next-generation sequencing reveals novel genomic and functional features. PLoS Pathog..

[42] Koopal S., Furuhjelm J.H., Jarviluoma A., Jaamaa S., Pyakurel P., Pussinen C., Wirzenius M., Biberfeld P., Alitalo K., Laiho M. (2007). Viral oncogene-induced DNA damage response is activated in kaposi sarcoma tumorigenesis. PLoS Pathog..

[43] Bryan B.A., Dyson O.F., Akula S.M. (2006). Identifying cellular genes crucial for the reactivation of kaposi’s sarcoma-associated herpesvirus latency. J. Gen. Virol..

[44] Ward I.M., Chen J. (2001). Histone H2AX is phosphorylated in an atr-dependent manner in response to replicational stress. J. Biol. Chem..

[45] Fernandez-Capetillo O., Chen H.T., Celeste A., Ward I., Romanienko P.J., Morales J.C., Naka K., Xia Z., Camerini-Otero R.D., Motoyama N. (2002). DNA damage-induced g2-m checkpoint activation by histone h2ax and 53bp1. Nat. Cell Biol..

[46] Bailey S.G., Verrall E., Schelcher C., Rhie A., Doherty A.J., Sinclair A.J. (2009). Functional interaction between epstein-barr virus replication protein zta and host DNA damage response protein 53bp1. J. Virol..

[47] Lilley C.E., Chaurushiya M.S., Boutell C., Everett R.D., Weitzman M.D. (2011). The intrinsic antiviral defense to incoming HSV-1 genomes includes specific DNA repair proteins and is counteracted by the viral protein ICP0. PLoS Pathog..

